# Motion error analysis of a shield machine tool-changing robot based on a screw-vector method

**DOI:** 10.1038/s41598-022-24847-6

**Published:** 2022-11-28

**Authors:** Wenxue Qian, Shuai Song, Kexin Liu, Xianhai Zeng, Xiaowei Yin, Liyang Xie

**Affiliations:** 1 School of Mechanical Engineering and Automation, Northeastern University, Shenyang, 110819 China; 2 Beijing Institute of Aerospace Control Devices, Beijing, 100039 China; 3 School of Mechanical, Shenyang Institute of Engineering, Shenyang, 110136 China; 4 Key Laboratory of Vibration and Control of Aero-Propulsion Systems Ministry of Education of China, Northeastern University, Shenyang, 110819 China

**Keywords:** Mechanical engineering, Civil engineering

## Abstract

Industrial robots are widely used in various industrial fields, such as handling and welding, due to their good repeat positioning accuracy. The motion error determines the absolute accuracy. For robot design, dimensional parameter errors and drive parameter errors, a mathematical model of a kinematic exponential product with error screws was proposed. The influence of different rod lengths and transmission errors on the accuracy of the end motion was analysed. A composite analysis method based on screw theory and vector method is proposed for the spatial deflection error of robot rotating joints with clearance. By using screw theory, a mathematical error model of the axial movement and spatial deflection of the joint gap was established. A mathematical model of joint space radial movement was established by using the three-dimensional vector method. Through numerical simulation, the position distribution law of the random error of the robot terminal in the workspace and the distribution of the plane projection density were obtained. By solving the attitude matrix, the distribution of each Euler angle error was obtained. A simulation test was carried out to verify the model's correctness. The calculation showed that the method is simple and correct, and the obtained error distribution characteristics are of great significance to improving robotic kinematic calibration accuracy and optimising the spatial position error distribution.

## Introduction

For the disc cutter changing technology of the slurry balanced shield machine, at present, the manual method is commonly used for disc cutter changing operation, and there are many dangers in the process of cutter changing, such as the ground collapse phenomenon will occur in the normal pressure disc cutter change method. Due to the large pressure difference, the tool changing with pressure will cause uncontrollable physical injury to the staff. Therefore, it is meaningful to realise automatic tool-changing for shield machines by a tool-changing robot. However, the motion accuracy of the tool-changing robot is very important for successful tool-changing.

Motion accuracy is an important technical index of a robot's ability to complete industrial tasks and its reliability in performing industrial tasks^1,2^. During static pose error analysis, the common factors affecting motion accuracy include original errors, manufacturing errors, control driving errors, and environmental errors^3,4^.

Robot static pose error modelling methods are divided into two main categories: the matrix method and the vector method^5,6^. The matrix method mainly uses the D–H linkage coordinate system to bring the error parameters into a homogeneous transformation matrix. Then, the terminal error is found using the transitivity of the kinematics. Yang et al.^7^ combined with the meta-action theory, studied the spatial structure relationships and motion transfer relationships of the series–parallel machine tool. Hu et al.^8^ established a positioning error model of a robot using a modified D–H model method with the JLRB20 industrial robot. They set the small error variables for the linkage parameters with the principle of small displacement error and made a differential change to the error model by combining kinematics. Finally, they solved the pose error distribution at the end of the robot. Ding et al.^9^ combined a new five-degree-of-freedom handling robot and projected the workspace to three planes parallel to the base coordinate system. Then, they obtained the specific parameters. With the D–H method, they established a positive kinematics model and solved it to obtain an inverse kinematics model. Finally, they achieved the maximum allowable error of each joint using the Jacobi matrix. Pan et al.^10^ established a kinematic model of a spraying robot body and a spray gun based on the screw method. They mainly analysed the rolling chain transmission error model. Combined with the kinematic parameters of the body, they established a motion error model of the spraying robot based on random variables, and experiments verified the correctness of the analysis method.

For the case of rotating joints with clearance, Yu et al.^11^ established a mechanism error analysis model based on the full differential force theory. Then, they effectively improved the convergence of the latest optimisation algorithm by introducing linearly decreasing inertia weights and compression factors. Zheng et al.^12^ used the spatial vector principle to establish radial and axial clearance models and analysed the allowable value of radial runout of the rotating joint. However, they did not consider the axial tilt phenomenon but only the axial length error. Finally, a clearance error model was established by combining the vector method.

However, the pose error models using the D-H parameter method all follow along the X-axis and Z-axis without considering the influence of the Y-axis. When two adjacent joint axes are parallel, any small error in the actual structural parameters will be sharply amplified due to the singularity of the model^13,14^. Many scholars have proposed new modelling methods in response to the above deficiencies. Jing et al.^15^ performed a matrix transformation based on a kinematic positive solution mapping of the motion screw and obtained each joint variable. Finally, a robot kinematics model was established using the screw formula and exponential product. Fu et al.^16^ proposed an efficient calibration method based on Lie theory to establish a unified error model of kinematic error and flexibility error. This method can simultaneously calibrate the kinematic parameters and joint flexibility of serial robots. The pose error of the terminal cutter is linearly superposed by the kinematic error and the flexibility error, and a unified error model was established. Huang et al.^17^ defined the position error screw and attitude error screw of the robot tool coordinate system, thus establishing a kinematic exponential product formula for a robot with the error screw. However, the error screw only considered the rotation angle error of the joint axes without considering the direction of the axis deflection in space. Chen et al.^18^ directly constructed an orthogonal partition matrix using the exponential product formula and established a kinematic error model that satisfies a parallel robot. Then, they proposed an analytical algorithm to determine and eliminate the redundant error components of passive joints. Moon et al.^19^ proposed a general mathematical framework to calculate and compensate for the terminal-operation error of the serial mechanism by using screw theory. Compared with the traditional D-H method, it simplifies the solution of the inverse kinematics and subsequent error compensation program. The method performs the error screw solution in the overall reference system instead of the tedious coordinate transformation in the local reference system.

The kinematic model can be built more conveniently by the exponential product method based on screw theory, and the method has been generalised^20,21^. The normal line can characterise the deviation between the nominal torsion angle of the central shaft and the actual shaft rotation angle. The offset and size error parameters of the connecting rod can be described by the spatial position vector of the joint centre. The normal line can describe the deflection of the axis. In summary, this study establishes the kinematic equations of a shield machine tool-changing robot with the screw method and proposes a pose error analysis model combining the screw and vector methods for rotating joints with clearance.

## Structure and working principle of the tool-changing robot

**Figure 1:**
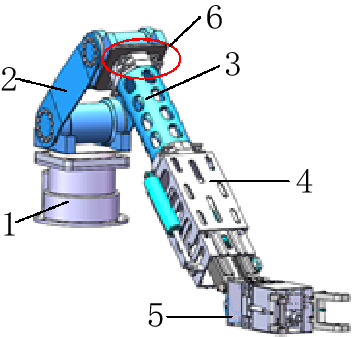
3D model of the tool-changing robot. 1: Base (the 1st joint), 2: Pitch joint (the 2nd and 3rd joint), 3: Extended arm, 4: Telescopic arm (the 4th joint), 5: End-effector, 6: Quick-change connection device.

**Figure 2:**
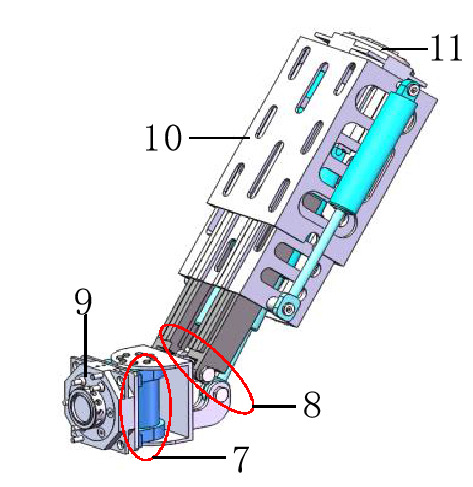
Schematic diagram of the telescopic rotating structure. 7: The 6th joint, 8: The 5th joint, 9: The male end of the quick-change connection device, 10: Telescopic arm, 11: The female end of the quick-change connection device.

The overall structure of the tool-changing robot is shown in Figs. 1 and 2, and it mainly consists of a base, a telescopic rotating structure, an end effector, and a quick-change connection device.

The base consists of a helical oscillating hydraulic cylinder, a base plate, and a connecting device to bear the weight of the whole body and support the movement of the whole robot, while the base is equipped with a first rotary joint to provide a steering function for the body, which can realise the overall left and right rotation of the tool-changing robot. The telescopic rotating structure mainly consists of a base arm, an extended arm, and a telescopic arm, which realise the function of telescoping, rotating and then achieving the purpose of changing the spatial attitude and position of the tool-changing robot. For different positions of the tool, a specific pose is formed to complete the tool change operation. The 5th and 6th joints are the pitch joint and the rotary joint. The two joints are mainly provided by the oscillating hydraulic cylinder, which can make the end-effector perform pitch and rotate functions. The application of joint 6 and joint 1 can ensure that the terminal working surface is parallel to the surface of the workpiece.

### Kinematic analysis

**Figure 3:**
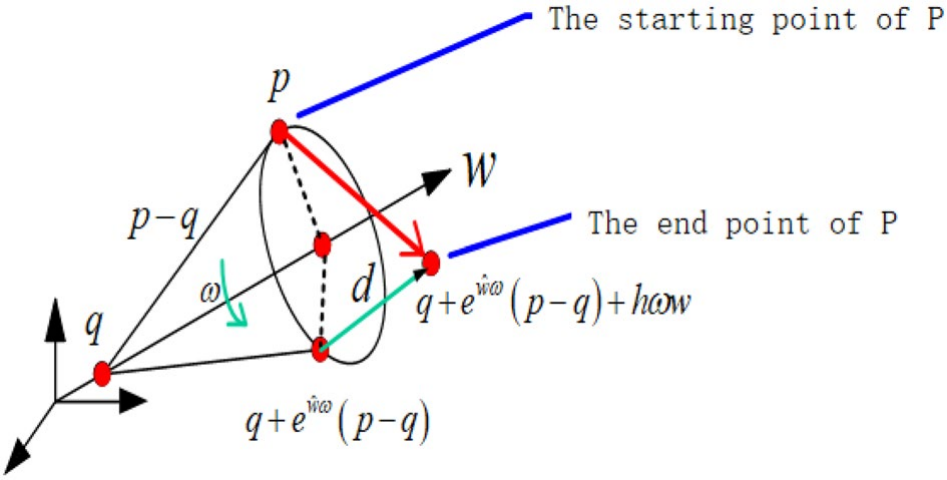
The decomposition diagram of screw motion.

According to the structure of the tool-changing robot, its kinematics can express the spatial poses of the terminal relative to the base coordinates through the transmission of each joint variable. The screw motion describes the motion change of the rigid body system from one pose state to another. The motion can be described as a composite motion of the rigid body rotating around a linear axis in space and moving along the line. As shown in Fig. 3, the rigid body motion is rotated through an angle $\omega$ around the axis of rotation in space and then moved along this axis by a distance of *d*.

The motion of the rigid body can be represented by the homogeneous transformation matrix ***G***, and ***G*** is determined by the position vector ${\varvec{p}} \in {\varvec{R}}^{3}$ and the attitude matrix ${\varvec{R}} \in {\varvec{SO}}\left( 3 \right)$ of the rigid body, namely:

1 $${\varvec{G}} = \left[ {\begin{array}{*{20}c} {\varvec{R}} & {\varvec{p}} \\ {\mathbf{0}} & 1 \\ \end{array} } \right] \in {\varvec{R}}^{4}$$

${\varvec{SO}}\left( 3 \right)$ is a particular orthogonal group containing attitude matrix ***R***, which is called a three-dimensional rotation group, expressed as:

2 $${\varvec{SO}}\left( 3 \right) = \left\{ {{\varvec{R}}|{\varvec{R}}^{{\text{T}}} {\varvec{R}} = I,\det \left( {\varvec{R}} \right) = \pm 1} \right\}$$

For moving joints, construct a motion screw for each joint:

3 $${\varvec{\xi}}_{i} = \left[ {\begin{array}{*{20}c} {\mathbf{0}} \\ {\varvec{v}} \\ \end{array} } \right] = \left[ {\begin{array}{*{20}c} {\mathbf{0}} \\ { - \hat{\user2{s}}_{i} \omega_{i} \times {\varvec{q}}_{i} + h_{i} \hat{\user2{s}}_{i} \omega_{i} } \\ \end{array} } \right] \in {\varvec{R}}^{6}$$

For rotating joints, construct a motion screw for each joint:

4 $${\varvec{\xi}}_{i} = \left[ {\begin{array}{*{20}c} {\varvec{w}} \\ {\varvec{v}} \\ \end{array} } \right] = \left[ {\begin{array}{*{20}c} {\hat{\user2{s}}_{i} \omega_{i} } \\ { - \hat{\user2{s}}_{i} \omega_{i} \times {\varvec{q}}_{i} + h_{i} \hat{\user2{s}}_{i} \omega_{i} } \\ \end{array} } \right] \in {\varvec{R}}^{6}$$

In the formula, ‘$w$’ is the angular velocity component, ‘$v$’ is the linear velocity component, ‘$\hat{\user2{s}}$’ is the unit vector of the joint axis, ‘$h$’ is the pitch, indicating the ratio of linear velocity to angular velocity, ‘$\omega$’ is the angular velocity and ${\varvec{q}} \in {\varvec{R}}^{3}$ is any point on the joint axis. The matrix format ${\varvec{\xi}}$ can be represented by $\hat{\user2{\xi }}$:

5 $$\hat{\user2{\xi }}_{i} = \left[ {\begin{array}{*{20}c} {\hat{\user2{w}}} & {\varvec{v}} \\ {\mathbf{0}} & 0 \\ \end{array} } \right] \in {\varvec{se}}\left( 3 \right)$$

In the formula, $\hat{w}$ is the antisymmetric matrix of $w$:

6 $$\hat{\user2{w}} = \left[ {\begin{array}{*{20}c} 0 & { - w_{z} } & {w_{y} } \\ {w_{z} } & 0 & { - w_{x} } \\ { - w_{y} } & {w_{x} } & 0 \\ \end{array} } \right] \in {\varvec{so}}\left( 3 \right)$$

When ${\varvec{\xi}} = \left( {{\varvec{w}},{\varvec{v}}} \right)$ is the screw axis, if $\left\| w \right\| = 1$, for any $\omega \in {\varvec{R}}$:

7 $$\begin{aligned} e^{{\hat{\user2{\xi }}\omega }} & = \left[ {\begin{array}{*{20}l} {e^{{\hat{\user2{w}}\omega }} } \hfill & {\left( {I - e^{{\hat{\user2{w}}\omega }} } \right)\left( {{\varvec{w}} \times {\varvec{v}}} \right) + {\varvec{ww}}^{T} {\varvec{v}}\omega } \hfill \\ {\mathbf{0}} \hfill & 1 \hfill \\ \end{array} } \right] \\ & = \left[ {\begin{array}{*{20}c} {\varvec{R}} & {\varvec{P}} \\ {\mathbf{0}} & 1 \\ \end{array} } \right]\left( {\omega \ne 0} \right) \\ \end{aligned}$$

Expanding $e^{{\hat{\user2{w}}\omega }}$ using the Rodrigues formula gives:

8 $$\begin{gathered} e^{{\hat{\user2{w}}\omega }} = I + \hat{\user2{w}}t + \frac{{\left( {\hat{\user2{w}}t} \right)^{2} }}{2!} + \frac{{\left( {\hat{\user2{w}}t} \right)^{3} }}{3!} + \cdots \hfill \\ \;\;\;\;\;\; = I + \hat{\user2{w}}\sin \omega + \hat{\user2{w}}^{2} \left( {1 - \cos \omega } \right) \hfill \\ \end{gathered}$$

The motion screw on the *i*th joint axis is $\xi_{i}$. According to the formula of the rigid body transformation matrix, we can obtain:

9 $${\varvec{g}}_{i,i}^{0} = \exp \left( {\hat{\user2{\xi }}_{1} ,{\varvec{\vartheta}}_{i,1} } \right) \cdots \exp \left( {\hat{\user2{\xi }}_{6} ,{\varvec{\vartheta}}_{i,6} } \right)$$

Additionally, $\omega_{i}$ can be divided into an initial position variable $\omega_{i,0}$ and a position increment $\omega_{i,\vartriangle }$. Then, Eq. (9) can be expressed as:

10 $$\begin{aligned} {\varvec{g}}_{i,i}^{0} \left( {\theta_{i} } \right) & = \prod\limits_{j = 1}^{6} {\exp \left( {\hat{\user2{\xi }}_{j} ,\vartheta_{i,j} } \right)\exp \left( {\hat{\user2{\xi }}_{j} ,\rho_{j} } \right)} \\ & { = }\;{\varvec{g}}_{i} \prod\limits_{j = 1}^{6} {\exp \left( {\hat{\user2{\xi }}_{j} ,\rho_{j} } \right)} \\ \end{aligned}$$

In the formula, $\vartheta_{i,6} = \omega_{i}$; $\rho_{i} = \omega_{i,\vartriangle }$.

${\varvec{g}}_{i}$ represents the initial pose of the {*i*}th coordinate system, which can be determined by the geometric shape of the connection and joint offset. Therefore, Eq. (10) is expressed by the first kind of normative coordinates as follows:

11 $${\varvec{g}}_{i} = \prod\limits_{j = 1}^{6} {\exp \left( {\hat{\user2{\xi }}_{j} ,\omega_{j} } \right)}$$

By setting ${\varvec{G}} = {\varvec{g}}_{i}$, the rigid body along this axis of motion screw ${\varvec{\xi}}_{i}$ when transformed from one pose to another by rotation and movement can be expressed as:

12 $${\varvec{T}}_{ab} \left( \omega \right) = {\varvec{G}} \cdot {\varvec{T}}_{ab} \left( 0 \right)$$

where ${\varvec{T}}_{ab} \left( 0 \right)$ represents the initial pose of the rigid body relative to the base coordinate system and ${\varvec{T}}_{ab} \left( \omega \right)$ represents the pose of a rigid body relative to the base coordinate system after transformation.

Set $\left\{ S \right\}$ as the base coordinate system and $\left\{ T \right\}$ as the terminal tool coordinate system, when $\omega = 0$ is the initial pose of the robot. Combining Eqs. (7), (8) and (12), the kinematic exponential product formula of the n-degree-of-freedom robot based on screw theory is obtained by combining the motion of each joint:

13 $${\varvec{T}}\left( \omega \right) = {\varvec{g}}_{1} {\varvec{g}}_{2} \cdots {\varvec{g}}_{n} {\varvec{T}}\left( 0 \right)$$

## Transmission error modelling and analysis

### Error modelling

The system error and transmission error of the robot can be regarded as the integration of tiny motion screws. After the integration of all error screws, the terminal motion error of the robot can be obtained through the transmission between adjacent rods.

**Figure 4:**
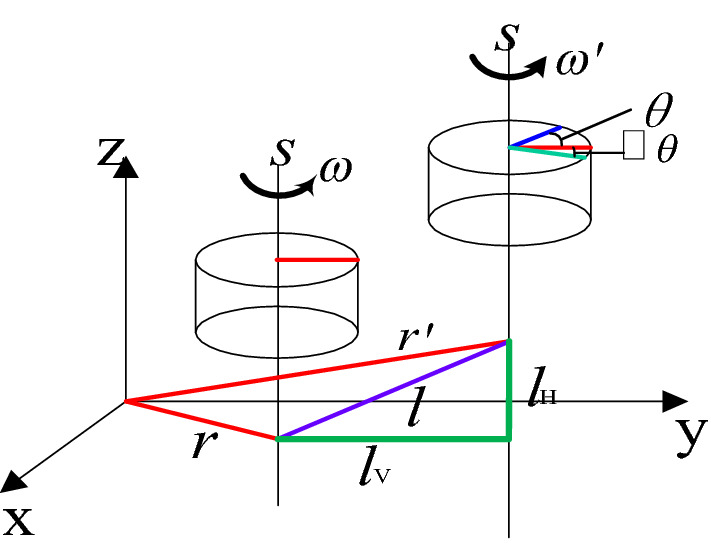
The schematic diagram of the error screw.

Figure 4 shows the error screw. The axis offset distance is *d*, and the error angle of joint transmission is $\vartriangle \omega$.

For rotating joints, the error is caused by the system error in the static state; then, the actual motion screw of a joint is:

14 $$\user2{\xi^{\prime}} = [\begin{array}{*{20}c} {\user2{v^{\prime}}} & {\user2{w^{\prime}}} \\ \end{array} ]^{T} = \left[ {\begin{array}{*{20}c} {\user2{r^{\prime}} \times {\varvec{w}}} & {\varvec{w}} \\ \end{array} } \right]^{T} \in {\varvec{R}}^{6 \times 1}$$

System error and transmission error will directly affect the form of the motion screw. The change is denoted as *q*, and then the error motion screw and the value of the motion can be expressed as:

15 $$\user2{\hat{\xi }^{\prime}}{ = }\left[ {\begin{array}{*{20}c} {\hat{\user2{w}}} & { - {\varvec{w}} \times \left( {{\varvec{q}} + \vartriangle {\varvec{q}}} \right)} \\ {\mathbf{0}} & 0 \\ \end{array} } \right]$$

16 $$\omega^{\prime}{ = }\int_{0}^{T} {\omega_{v} \cdot \prod\limits_{j = 1}^{n} {\Re_{j} dt} }$$

$\hat{\xi }^{\prime}$ where is the actual screw of the screw unit axis $\hat{\xi }$, $\omega^{\prime}$ is the actual motion variable value, $\omega_{v}$ is the ideal motion velocity of the screw unit axis $\hat{\xi }$, *T* is the motion time, and $\Re_{j}$ is the error transmission index between adjacent motion screws.

For translational motion joints, the system error and transmission error will also directly affect the form of the motion screw. The actual motion screw of a certain joint is:

17 $$\xi^{\prime} = [\begin{array}{*{20}c} {v^{\prime}} & 0 \\ \end{array} ]^{T} = \left[ {\begin{array}{*{20}c} {r^{\prime} \times w^{\prime}} & 0 \\ \end{array} } \right]^{T} \in R^{6 \times 1}$$

When the translation of the unit screw axis occurs, the change is denoted as $\vartriangle w$; then, the error motion screw and motion value can be expressed as:

18 $$\hat{\xi }^{\prime}{ = }\left[ {\begin{array}{*{20}c} 0 & {\frac{v + \vartriangle v}{{\left| {v + \vartriangle v} \right|}}} \\ 0 & 0 \\ \end{array} } \right]$$

19 $$\omega^{\prime}{ = }\int_{0}^{T} {v_{v} \cdot \prod\limits_{j = 1}^{n} {\Re_{j} dt} }$$

In the formula,$v_{v}$ is the ideal motion speed of the translational screw axis.

The Plucker coordinate transformation can express the motion process of the joint from the ideal axis to the actual axis, which is regarded as the motion result of $\omega_{e} \xi_{e}$.$\omega_{e}$ is the magnitude of the error screw. According to Eq. (9), the error screw is:

20 $${\varvec{\xi}}_{e} = \left[ {\begin{array}{*{20}c} {{\varvec{v}}_{e} } & {{\varvec{w}}_{e} } \\ \end{array} } \right]^{T} = \left[ {\begin{array}{*{20}c} {\user2{r^{\prime}} \times {\varvec{w}}_{e} } & {{\varvec{w}}_{e} } \\ \end{array} } \right]^{T}$$

${\varvec{w}}_{e}$ is the unit vector of the axis deflection direction.

Therefore, under the action of system error, the actual pose of the end effector relative to the base coordinate system is:

21 $$\user2{T^{\prime}}\left( \omega \right) = e^{{\omega_{e1} \hat{\user2{\xi }}_{e1} }} e^{{\omega_{e2} \hat{\user2{\xi }}_{e2} }} \cdots e^{{\omega_{en} \hat{\user2{\xi }}_{en} }} {\varvec{T}}\left( 0 \right)$$

When considering the transmission error, the robot kinematics exponential product model is:

22 $${\varvec{T}}\left( \omega \right) = e^{{\omega_{e1} \hat{\xi }_{e1} }} e^{{(\omega_{1} + \vartriangle \omega_{1} )\hat{\xi }_{1} }} e^{{\omega_{2} \hat{\xi }_{2} }} \cdots e^{{\omega_{en} \hat{\xi }_{en} }} e^{\begin{subarray}{l} (\omega_{n} + \vartriangle \omega_{n} ) \\ 0\hat{\xi }_{n} \end{subarray} } {\varvec{T}}\left( 0 \right)\;\;\;\left( {n = 1,2,3, \ldots ,6} \right)$$

In the formula, $\omega_{ei}$ is the magnitude of the error screw; $\xi_{ei}$ is the unit error screw; $\omega_{i}$ is the ideal value of joint motion, and $\xi_{i}$ is the ideal unit screw of the joint.

**Figure 5:**
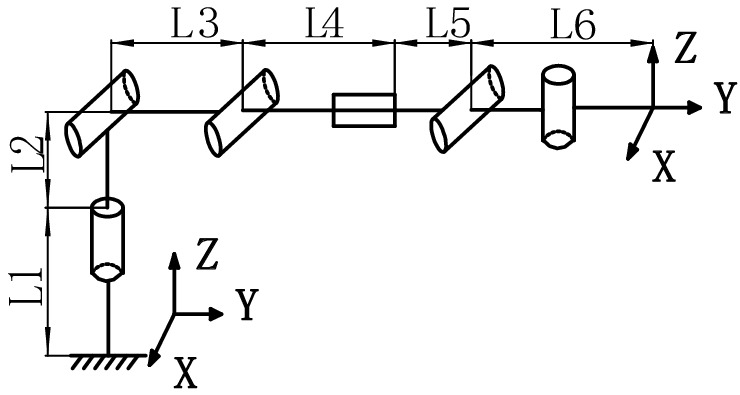
Simplified structure and coordinate system of the robot.

Establishing the robot coordinate system shown in Fig. 5, the terminal poses are the same as the base coordinate system. The terminal poses in the ideal state are:

23 $${\varvec{T}}\left( {0} \right){ = }\left[ {\begin{array}{*{20}c} {1} & {0} & {0} & 0 \\ {0} & {1} & {0} & {l_{3} + l_{4} + l_{5} + l_{6} } \\ {0} & {0} & {1} & {l_{1} + l_{2} } \\ {0} & {0} & {0} & {0} \\ \end{array} } \right]$$

When considering the size error, the position vector from the point above the rotating shaft to the origin of the base coordinate system is selected as:

24 $$\left\{ {\begin{array}{*{20}l} {\user2{r^{\prime}}_{1} = \left[ {\begin{array}{*{20}c} 0 & 0 & {l_{1} + \vartriangle l_{1} } \\ \end{array} } \right]^{T} } \hfill \\ {\user2{r^{\prime}}_{2} = \left[ {\begin{array}{*{20}c} 0 & 0 & {l_{1} + \vartriangle l_{1} + l_{2} + \vartriangle l_{2} } \\ \end{array} } \right]^{T} } \hfill \\ {\user2{r^{\prime}}_{3} = \left[ {\begin{array}{*{20}c} 0 & {l_{3} + \vartriangle l_{3} } & {l_{1} + \vartriangle l_{1} + l_{2} + \vartriangle l_{2} } \\ \end{array} } \right]^{T} } \hfill \\ {\user2{r^{\prime}}_{4} = \left[ {\begin{array}{*{20}c} 0 & {l_{3} + \vartriangle l_{3} + l_{4} + \vartriangle l_{4} } & {l_{1} + \vartriangle l_{1} + l_{2} + \vartriangle l_{2} } \\ \end{array} } \right]^{T} } \hfill \\ {\user2{r^{\prime}}_{5} = \left[ {\begin{array}{*{20}c} 0 & {l_{3} + \vartriangle l_{3} + l_{4} + \vartriangle l_{4} + l_{5} + \vartriangle l_{5} } & {l_{1} + \vartriangle l_{1} + l_{2} + \vartriangle l_{2} } \\ \end{array} } \right]^{T} } \hfill \\ {\user2{r^{\prime}}_{6} = \left[ {\begin{array}{*{20}c} {0} & {l_{3} + \vartriangle l_{3} + l_{4} + \vartriangle l_{4} + l_{5} + \vartriangle l_{5} + l_{5} + \vartriangle l_{6} } & {l_{1} + \vartriangle l_{1} + l_{2} + \vartriangle l_{2} } \\ \end{array} } \right]^{T} } \hfill \\ \end{array} } \right.$$

In the motion process, the rotation error around the z-axis is $\vartriangle \omega_{i}$, and the magnitude of the motion screw of each joint axis is $\omega_{i} + \vartriangle \omega_{i}$.

The kinematic model of the robot with error screws is obtained by substituting the above formula into Eq. (13).

According to the mechanical design criteria and dimensional machining requirements, the error distribution is set as shown in Table 1.

**Table 1:** The distribution of each error.

Error type	Distribution type	Mean value	Standard deviation
Linkage dimension error	Normal distribution	0 mm	0.2 mm
Driving error of moving joint	Normal distribution	0 mm	0.3 mm
Driving error of rotating joint	Normal distribution	0 rad	0.0087 rad
Joint axis deflection error	Normal distribution	0 rad	0.00435 rad

### Simulation analysis

To analyse the influence of errors on the kinematic accuracy of the robot terminal, 20,000 random data were generated for each error using the Monte Carlo sampling method, and each group of data conforms to the distribution shown in Table 1. Table 2 lists the robot linkage parameters.

**Table 2:** Error distribution table of the tool-changing robot.

Linkage number	Mean value of linkage length	Standard deviation of linkage length error
1	315 mm	0.17 mm
2	210 mm	0.15 mm
3	796 mm	0.2 mm
4	560 mm	0.25 mm
5	240 mm	0.15 mm
6	165 mm	0.15 mm

Joint number	Mean value of motion	Standard deviation of motion error
1	15π/180 rad	0.3 deg
2	30π/180 rad	0.3 deg
3	− 10π/180 rad	0.1548 deg
4	0 mm	0.25 mm
5	− 20π/180 rad	0.5 deg
6	− 5π/180 rad	0.3 deg

The spatial position of the end effector in the ideal state is [− 0.0924, 0.1609, − 1.5119]. For the error of each group obeying a normal distribution, each data point is brought into Eq. (11), and the actual spatial pose coordinates can be obtained.

**Figure 6:**
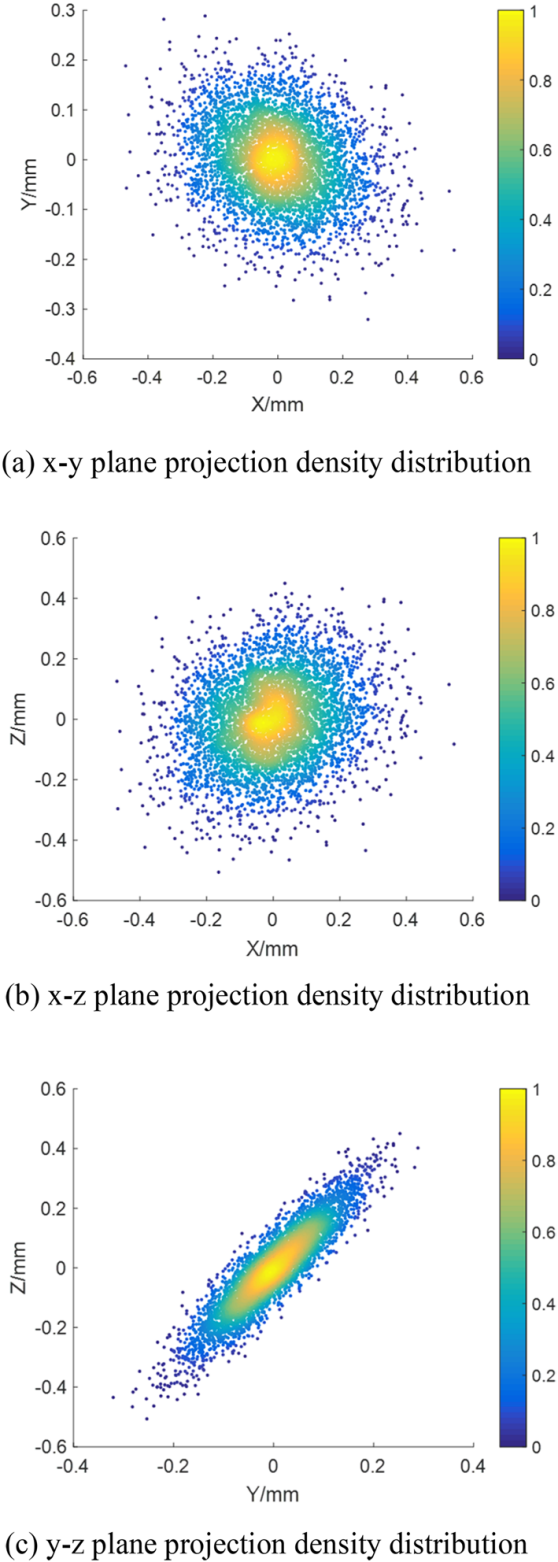
Error of three-dimensional spatial distribution and plane projection density distribution. (**a**) x–y plane projection density distribution, (**b**) x–z plane projection density distribution, (**c**) y–z plane projection density distribution.

Figure 6 shows that the projection density value of the three planes is relatively large near (0, 0), and the projection density gradually decreases and spreads around. This indicates that the error distribution is concentrated near the ideal state, and its reliability is high.

Table 3 shows that the dimension error of the connecting rod has a great influence on the spatial position error. The mean value error is 0.0112 mm, and the standard deviation is 0.1105 mm. Among the errors in the three directions, the most influential ones are the y and z directions, the mean value is close to zero, and the standard deviations are 0.0822 mm and 0.1414 mm.

**Table 3:** Numerical characteristics of terminal position error of the tool-changing robot.

Dependent variable	Mean value	Standard deviation	Maximum value	Minimum value
Δ*p*/mm	0.0112	0.1105	0.4754	− 0.3804
Δ*x*/mm	− 4.71 × 10^–5^	0.1389	0.5672	− 0.6406
Δ*y*/mm	1.59 × 10^–4^	0.0822	0.2991	− 0.3574
Δ*z*/mm	2.54 × 10^–4^	0.1414	0.5389	− 0.6547

By setting the terminal position error of the tool-changing robot as T and attitude error as R, the total number of samples as $\lambda$, the total number of samples of position error as $\lambda_{P}$, and the total number of samples of attitude error as $\lambda_{\phi }$, the reliability of the welding robot mechanism pose is:

25 $$\left\{ \begin{gathered} P(\left| {\Delta P_{i} } \right| < R) = \lambda_{p} /\lambda \hfill \\ P(\left| {\Delta \phi_{i} } \right| < T) = \lambda_{{\phi_{i} }} /\lambda \hfill \\ \end{gathered} \right.$$

Therefore, the motion accuracy of the tool-changing robot in the working position based on screw theory is:

26 $$\begin{gathered} \left\{ {\begin{array}{*{20}l} {{\varvec{P}}\left( {\left| {\Delta {\varvec{P}}} \right| < {\varvec{T}}} \right) = 0.7639,\;\;\;\;\;\;T = 0.15\;{\text{mm}}} \hfill \\ {{\varvec{P}}\left( {\left| {\Delta {\varvec{P}}} \right| < {\varvec{T}}} \right) = 0.9503,\;\;\;\;\;\;T = 0.25\;{\text{mm}}} \hfill \\ \end{array} } \right. \hfill \\ {\varvec{P}}\left( {\left| {\Delta {\varvec{P}}} \right| < {\varvec{R}}} \right) = (0.7639,0.8458,0.9855),\;\;\;\;\;\;R = 0.002\;{\text{rad}} \hfill \\ \end{gathered}$$

**Figure 7:**
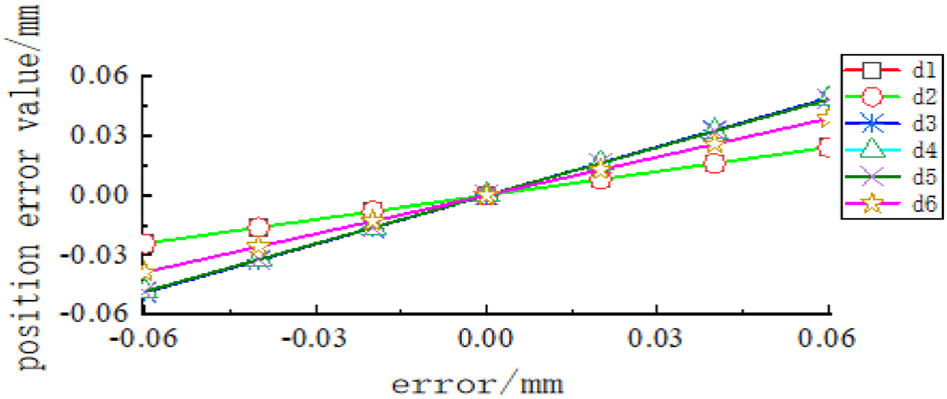
Sensitivity curve of linkage length error to terminal position error.

**Figure 8:**
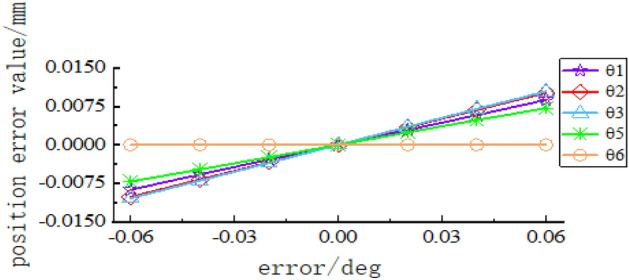
Sensitivity curve of rotation angle error to terminal position error.

**Figure 9:**
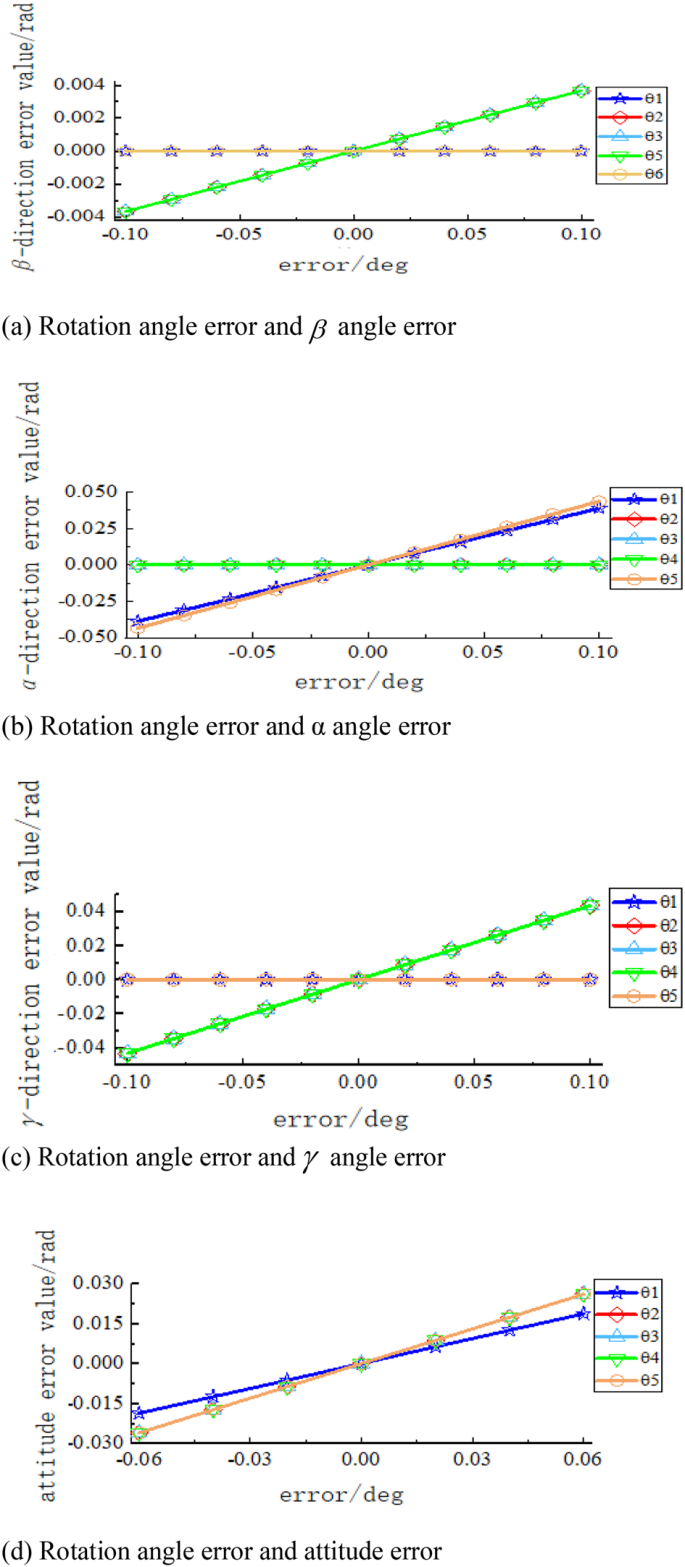
Sensitivity curve of rotation angle error to terminal attitude error. (**a**) Rotation angle error and $\beta$ angle error, (**b**) Rotation angle error and α angle error, (**c**) Rotation angle error and $\gamma$ angle error, (**d**) Rotation angle error and attitude error.

The control variable method was used to analyse the influence of the error model based on screw theory on the pose error at the end of the tool-changing robot. The linkage length error is set to $$\pm$$ 0.02 mm, $$\pm$$ 0.04 mm, and $$\pm$$ 0.06 mm, and the angle error is set to $$\pm$$$$0.02^\circ$$, $$\pm$$$$0.04^\circ$$, $$\pm$$$$0.06^\circ$$, $$\pm$$$$0.08^\circ$$ and $$\pm$$$$0.1^\circ$$. The sensitivity curves of the terminal error on the linkage length and angle error are shown in Figs. 7, 8 and 9 are obtained.

In Figs. 7, 8 and 9, it can be seen that the spatial position error has a greater sensitivity to the linkage length error of linkage 3 and linkage 4, as well as to the rotation angle error of joint 2 and joint 3. The joint rotation angle errors $\theta_{1}$ and $\theta_{2}$ have no effect on the $\beta$ angle error in the X–Y–Z fixed coordinate system. $\theta_{2} ,\theta_{3} ,\theta_{6}$ has the greatest effect on the $\beta$ angle error; $\theta_{1} ,\theta_{6}$ has a greater effect on the $\alpha$ angle error, of which the greatest effect is on the $\theta_{6}$ angle;$\theta_{2} ,\theta_{3} ,\theta_{5}$ has a greater effect on the $\gamma$ angle error, and the effect of all three is approximately the same. When there is a rotation error alone in each rotation angle, $\theta_{2} ,\theta_{3} ,\theta_{5} ,\theta_{6}$ has the greatest effect on the terminal attitude error, but the effect is minimal, which can be ignored in the subsequent analysis.

## Modelling and analysis of joint clearance error

### Error modelling

The 6-DOF serial tool-changing robot consists of five rotating joints and one moving joint. In the presence of error, when the centreline of the journal rotates around and moves along a straight line in space, the axial movement and spatial deflection error of the joint clearance can be regarded as a tiny motion screw integration.

**Figure 10:**
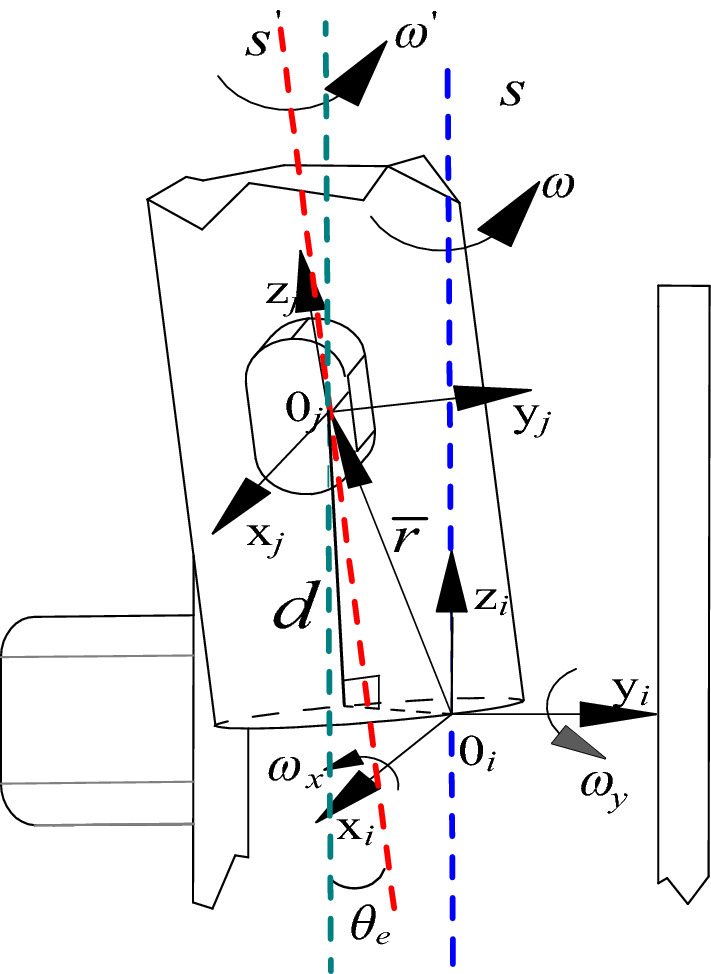
Schematic diagram of the error screw of axial movement and spatial deflection.

Figure 10 shows a schematic diagram of the error screw of axial movement and spatial deflection. It is known that the axis deflection angle is $\theta_{e}$, and the axial offset distance of the journal along the sleeve centreline is *d*.

For the rotating joint, the error screw coordinate of the screw unit axis $\xi$ is:

27 $${\varvec{\xi}}_{s} = \left[ { - {\varvec{\omega}}_{s} \times {\varvec{q}}_{s} \;\;\;\;{\varvec{\omega}}_{s} } \right]^{T} \in {\varvec{R}}^{6 \times 1}$$

The joint clearance error can directly affect the form of the motion screw, and the rotation axis can also change, which changes the relative spatial pose between the joints. Therefore, the change is denoted as $\vartriangle w$ and $\vartriangle q$. Then, the error motion screw and motion value can be expressed as:

28 $$\left\{ {\begin{array}{*{20}l} {\user2{\hat{\xi }^{\prime}}_{s} = \left[ {\begin{array}{*{20}c} {\left( {\frac{{\omega_{s} + \vartriangle \omega_{s} }}{{\left| {\omega_{s} + \vartriangle \omega_{s} } \right|}}} \right)} & {\frac{{ - \left( {\omega_{s} + \vartriangle \omega_{s} } \right)}}{{\left| {\omega_{s} + \vartriangle \omega_{s} } \right|}} \times \left( {q + \vartriangle q} \right)} \\ 0 & 0 \\ \end{array} } \right]} \hfill \\ {\omega^{\prime}_{s} = \int_{0}^{T} {\omega_{v} \cdot \prod\limits_{j = 1}^{n} {\Re_{j} dt} } } \hfill \\ \end{array} } \right.$$

For the translational joints, the error screw coordinate of the screw unit axis $\xi$ is:

29 $${\varvec{\xi}}_{s} { = }\left[ {\begin{array}{*{20}c} { - {\varvec{\omega}}_{s} \times {\varvec{q}}_{s} } & {\mathbf{0}} \\ \end{array} } \right]^{T} \in {\varvec{R}}^{6 \times 1}$$

In the translational joint, since the journal has only translation error and axis deflection motion error relative to the sleeve, the error motion screw and motion value can be expressed as:

30 $$\left\{ {\begin{array}{*{20}l} {\hat{\xi }^{\prime}_{s} = \left[ {\begin{array}{*{20}c} 0 & {\frac{{ - \left( {\omega_{s} + \vartriangle \omega_{s} } \right)}}{{\left| {\omega_{s} + \vartriangle \omega_{s} } \right|}} \times \left( {q + \vartriangle q} \right)} \\ 0 & 0 \\ \end{array} } \right]} \hfill \\ {\omega^{\prime}_{s} = \int_{0}^{T} {v_{v} \cdot \prod\limits_{j = 1}^{n} {\Re_{j} dt} } } \hfill \\ \end{array} } \right.$$

The joint movement from the ideal axis to the actual axis is regarded as the result of the joint movement of $\theta_{e} \xi_{e}$ and $r^{\prime}$_._$\theta_{e} \xi_{e}$ represents the deflection of the joint axis in space and $r^{\prime}$ represents the axial movement of the joint axis. Therefore, the error screw is:

31 $${\varvec{\xi}}_{e} = \left[ {\begin{array}{*{20}c} {{\varvec{w}}_{e} } & {{\varvec{v}}_{e} } \\ \end{array} } \right] = \left[ {\begin{array}{*{20}c} {{\varvec{w}}_{e} } & {\user2{r^{\prime}} \times {\varvec{w}}_{e} } \\ \end{array} } \right]^{T}$$

In the formula, ${\varvec{w}}_{e} = R_{ex}$, $R_{ey}$, or $R_{ez}$. $w_{e}$ is the unit direction vector of the joint deflection axis. It represents the rotation of the joint axis around the x-, y-, or z-axis of the base coordinate system, which realises the spatial deflection of the joint axis; therefore, the joint axis vector can be obtained by rotating the joint axis around the axis of the base coordinate system:

32 $$\user2{w^{\prime}} = Rot\left( {x,\theta_{ex} } \right)Rot\left( {y,\theta_{ey} } \right)Rot\left( {z,\theta_{ez} } \right){\varvec{w}}$$

Therefore, under the action of joint clearance, the actual pose of the terminal relative to the axial motion of the base coordinate system and the deflection around the centre point can be expressed in terms of the kinematic exponential product mode as:

33 $${\varvec{T}}\left( \theta \right) = e^{{\theta_{e1} \hat{\user2{\xi }}_{e1} }} e^{{\theta_{e2} \hat{\user2{\xi }}_{e2} }} \cdots e^{{\theta_{en} \hat{\user2{\xi }}_{en} }} {\varvec{T}}\left( 0 \right)$$

According to the schematic diagram of the robot structure in Fig. 5, the unit direction vector of each joint axis relative to the base coordinate system is:

34 $$\left\{ {\begin{array}{*{20}l} {{\varvec{w}}_{1} = {\varvec{w}}_{6} = [\begin{array}{*{20}c} 0 & 0 & 1 \\ \end{array} ]} \hfill \\ {{\varvec{w}}_{2} = {\varvec{w}}_{3} = {\varvec{w}}_{5} = \left[ {\begin{array}{*{20}c} 1 & 0 & 0 \\ \end{array} } \right]} \hfill \\ \end{array} } \right.$$

According to the direct distribution of the robot joint axis, the unit vector distribution of the rotation axis for each joint can be obtained as follows:

35 $$\left\{ {\begin{array}{*{20}l} {{\varvec{w}}_{ex} = \left[ {\begin{array}{*{20}c} 1 & 0 & 0 \\ \end{array} } \right],\;\;\;({\text{joint}}\;1,6)} \hfill \\ {{\varvec{w}}_{ey} = \left[ {\begin{array}{*{20}c} 0 & 1 & 0 \\ \end{array} } \right],\;\;\;({\text{joint}}\;1,{2},{3},{5},6)} \hfill \\ {{\varvec{w}}_{ez} = \left[ {\begin{array}{*{20}c} 0 & 0 & 1 \\ \end{array} } \right],\;\;\;({\text{joint}}\;{2},{3},{5})} \hfill \\ \end{array} } \right.$$

By the unit vector of the axis deflection around the base coordinate system, the antisymmetric matrix of the rotation axis unit vector can be obtained by combining with Eq. (3):

$$\hat{\user2{w}}_{ex} = \left[ {\begin{array}{*{20}c} 0 & 0 & 0 \\ 0 & 0 & { - 1} \\ 0 & 1 & 0 \\ \end{array} } \right],\;\;\;\hat{\user2{w}}_{ey} = \left[ {\begin{array}{*{20}c} 0 & 0 & 1 \\ 0 & 0 & 0 \\ { - 1} & 0 & 0 \\ \end{array} } \right],\;\;\;\hat{\user2{w}}_{ez} = \left[ {\begin{array}{*{20}c} 0 & { - 1} & 0 \\ 1 & 0 & 0 \\ 0 & 0 & 0 \\ \end{array} } \right]$$

Combined with the antisymmetric matrix of the unit vector, the spatial attitude of the joint axis relative to the base coordinate system after spatial deflection can be calculated by the Rodrigues formula:

36 $$e^{{\hat{\user2{w}}_{ei} \theta_{en} }} = I + \hat{\user2{w}}_{ei} \sin \theta_{en} + \hat{\user2{w}}_{ei}^{2} \left( {1 - \cos \theta_{en} } \right)$$

In the formula, $i = x,y,z;n = 1,2,3,5,6$

After the joint axis is rotated around the unit rotation axis, the spatial unit vector of the joint axis can be obtained as shown in the following formula:

37 $$\user2{w^{\prime}} = Rot(x,\theta_{ex} )Rot\left( {y,\theta_{ey} } \right)Rot\left( {z,\theta_{ez} } \right){\varvec{\omega}}$$

Therefore, the spatial unit vector of the joint axis is as follows:

39 $$\begin{gathered} \user2{w^{\prime}}_{m} = \left[ {\sin \theta_{ey}^{m} ; - \sin \theta_{ex}^{m} \cos \theta_{ey}^{m} ;\cos \theta_{ex}^{m} \cos \theta_{ey}^{m} } \right]^{T} \hfill \\ \user2{w^{\prime}}_{n} = \left[ { - \cos \theta_{ey}^{n} \sin \theta_{ez}^{n} ;\cos_{ez}^{n} ;\sin_{ey}^{n} \sin_{ez}^{n} } \right]^{T} \hfill \\ \end{gathered}$$

In the formula, $m = 1,6;\;n = 2,3,5$

When the axial movement error of clearance exists, the axial movement error is considered as the offset error along the unit director of the rotation axis for each joint at the ideal position. The position vector from the centre point above the rotating axis to the origin point of the base coordinate system is:

40 $$\left\{ \begin{gathered} \user2{r^{\prime}}_{1} = \left[ {0;0;l_{1} + \vartriangle l_{1} } \right] \hfill \\ \user2{r^{\prime}}_{2} = \left[ {0;\vartriangle l_{2} ;l_{1} { + }l_{2} + \vartriangle l_{1} } \right] \hfill \\ \user2{r^{\prime}}_{3} = \left[ {l_{3} ;\vartriangle l_{{3}} + \vartriangle l_{2} ;l_{1} { + }l_{2} + \vartriangle l_{1} } \right] \hfill \\ \user2{r^{\prime}}_{4} = \left[ {l_{4} + l_{3} ;\vartriangle l_{{3}} + \vartriangle l_{2} ;l_{1} { + }l_{2} + \vartriangle l_{1} } \right] \hfill \\ \user2{r^{\prime}}_{5} = \left[ {l_{4} + l_{3} + l_{5} ;\vartriangle l_{5} + \vartriangle l_{{3}} + \vartriangle l_{2} ;l_{1} { + }l_{2} + \vartriangle l_{1} } \right] \hfill \\ \user2{r^{\prime}}_{6} = \left[ {l_{4} + l_{3} + l_{5} + l_{6} ;\vartriangle l_{5} + \vartriangle l_{{3}} + \vartriangle l_{2} ;l_{1} { + }l_{2} + \vartriangle l_{1} + \vartriangle l_{6} } \right] \hfill \\ \end{gathered} \right.$$

${\varvec{v}}_{i}^{\prime } = - \user2{w^{\prime}}_{i} \times \user2{r^{\prime}}_{i}$, and from Eq. (5), the error screw can be obtained as:

41 $${\varvec{\xi}}_{ei} = \left[ {\begin{array}{*{20}c} {{\varvec{w}}_{ei}^{\prime } } & {\user2{v^{\prime}}_{ei} } \\ \end{array} } \right]\;\;\;\;\left( {i = 1,2,3,5,6} \right)$$

By substituting the above formula into Eqs. (6) and (7), the kinematic model of axial movement and spatial deflection with joint clearance error screw is obtained.

**Figure 11:**
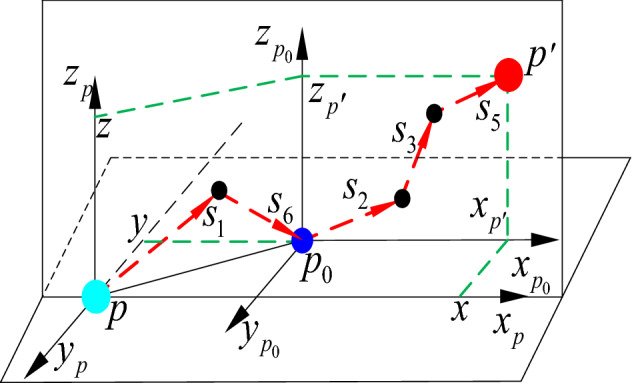
Three-dimensional spatial joint clearance error coordinate system.

When the axial movement error of clearance exists, the axial movement error is regarded as a massless rod (clearance rod). According to the axial distribution of each joint of the tool-changing robot, the radial error model of joint clearance is established by using the double plane analysis method, as shown in Fig. 11.

$s_{1}$ and $s_{6}$ move along the horizontal plane $x_{p} - y_{p}$.$s_{2} ,s_{3}$ and $s_{5}$ move along the vertical plane $x_{p} - z_{p}$. Point *p* is the starting point of the first group of vectors (ideal position point), the point $p_{0}$ is the starting point of the third group of vectors (moving terminal point in the horizontal plane), and two points are the origin of the coordinate system. Therefore, the point $p^{\prime}$ is the actual spatial position point. After continuous transformation in the coordinate systems $\left\{ p \right\}$ and $\left\{ {p_{0} } \right\}$, the spatial position (error position) of the point $p^{\prime}$ relative to point *p* can be obtained:

42 $$\left\{ {\begin{array}{*{20}l} {x_{{p^{\prime}}} = \sum\limits_{n} {s_{n} \cos \theta_{n} ,\left( {n = 1,2,3,5,6} \right)} } \hfill \\ {y_{{p^{\prime}}} = \sum\limits_{m} {s_{m} \sin \theta_{m} ,\left( {m = 1,6} \right)} } \hfill \\ {z_{{p^{\prime}}} = \sum\limits_{n} {s_{o} \sin \theta_{o} ,\left( {o = 2,3,5} \right)} } \hfill \\ \end{array} } \right.$$

In the formula, ${\varvec{s}}$ is the clearance rod vector. Therefore, $$p^{\prime} = \left[ {\begin{array}{*{20}c} {x_{{p^{\prime}}} } & {y_{{p^{\prime}}} } & {z_{{p^{\prime}}} } \\ \end{array} } \right]^{T}$$ is the radial motion spatial error value of the joint clearance.

### Simulation analysis

Considering the axial, radial movement and spatial deflection, the actual pose $p^{\prime}$ of the end effector can be obtained by the error model in the previous section:

43 $$p^{\prime}\left( \psi \right) = T\left( \theta \right) + W\left( \tau \right)$$

In the formula, $$W\left( \tau \right) = \left[ {\begin{array}{*{20}c} \varepsilon & {p^{\prime}} \\ {\varvec{0}} & 0 \\ \end{array} } \right]$$,$\varepsilon { = 0}_{{{3} \times {3}}}$.

The Monte Carlo random sampling method was used to set the axial movement distance of the joint as $d_{i}$, the length of the clearance rod in radial movement as $s_{i}$, the deflection angle (based on the last vector direction of the clearance rod) as $\theta_{i}$, and the deflection angles of the x-axis and y-axis in spatial deflection as $\omega_{xi}$ and $\omega_{yi}$. The specific parameter forms are shown in Table 4 and $\theta_{i}$ obeys the $\left[ {0,2\pi } \right]$ uniform distribution.

**Table 4:** Kinematics parameter values of joint clearance.

Parameter	Mean value	Standard deviation
$d_{i}$	0 mm	0.2 mm
$s_{i}$	0 mm	0.5 mm
$\omega_{xi}$	0 rad	0.087 rad
$\omega_{yi}$	0 rad	0.087 rad

**Figure 12:**
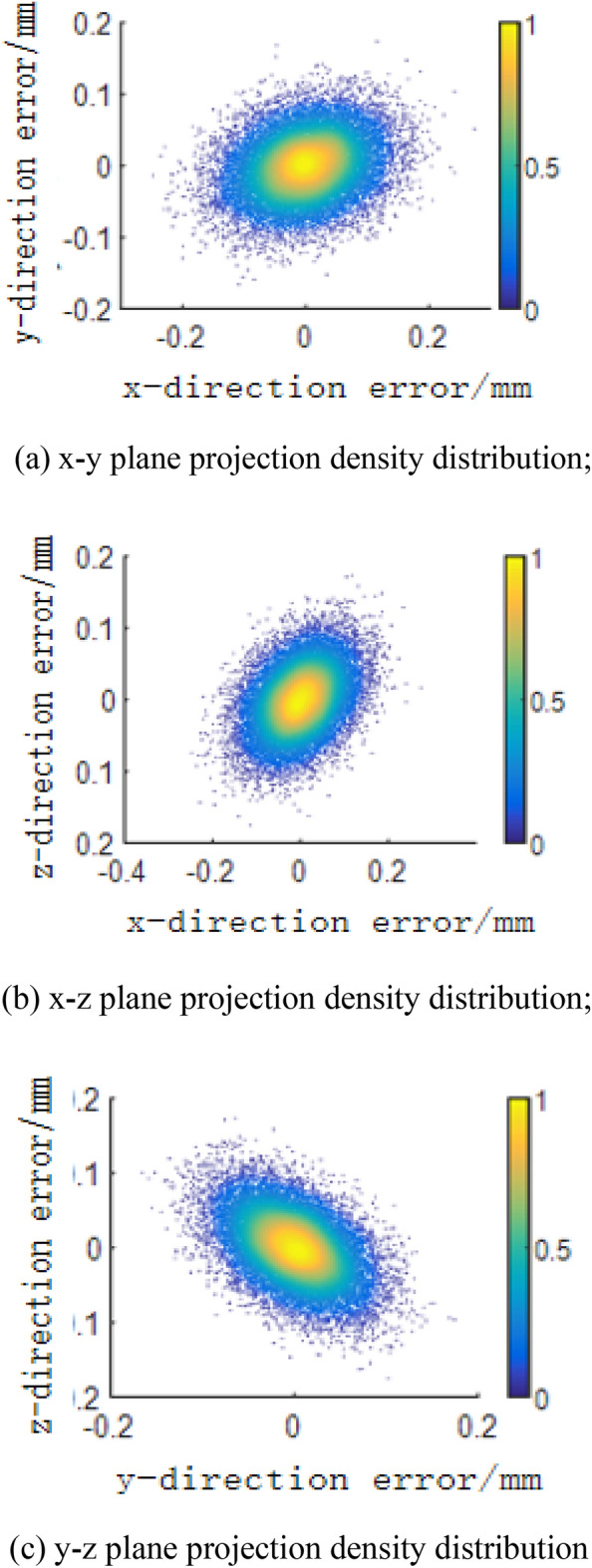
Error three-dimensional spatial distribution and plane projection density distribution. (**a**) x–y plane projection density distribution; (**b**) x–z plane projection density distribution; (**c**) y–z plane projection density distribution.

Figure 12 shows the distribution of error values in three-dimensional space and the projection density distribution of terminal error values in each plane; the error values are concentrated at 0 mm in the x-direction, y-direction and z-direction.

**Figure 13:**
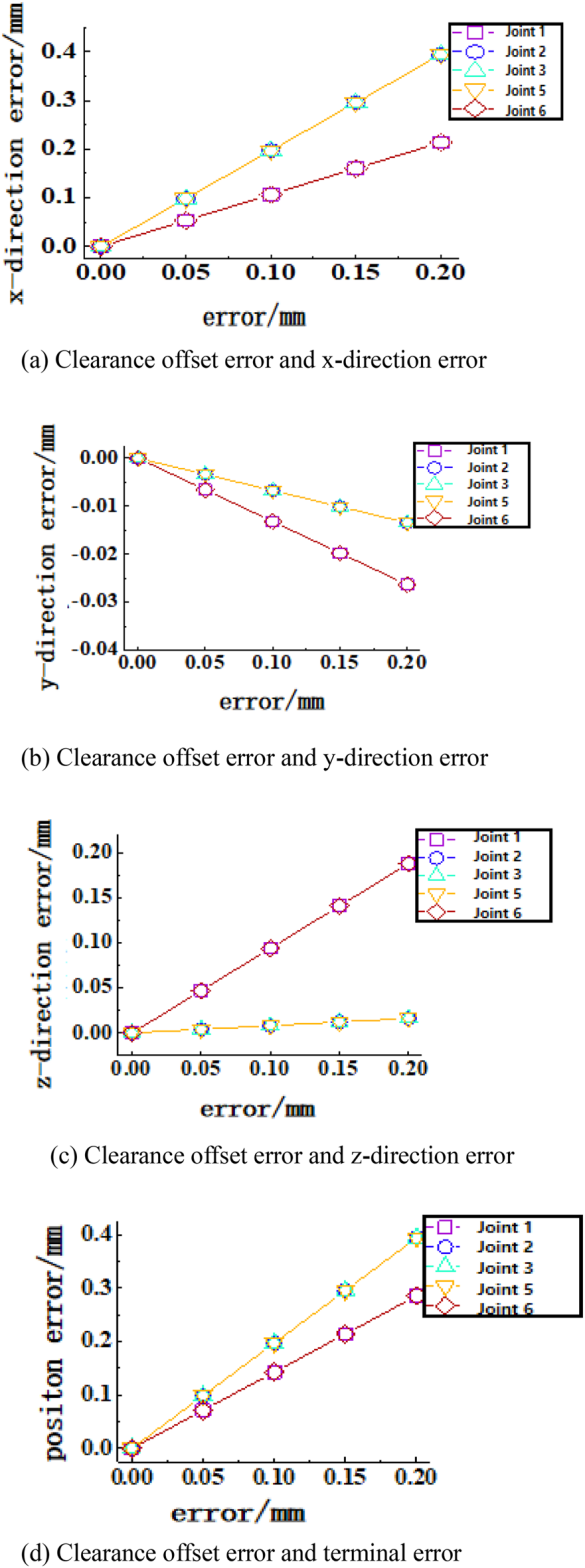
Joint clearance offset error-terminal position sensitivity curve. (**a**) Clearance offset error and x-direction error, (**b**) Clearance offset error and y-direction error, (**c**) Clearance offset error and z-direction error, (**d**) Clearance offset error and terminal error.

**Figure 14:**
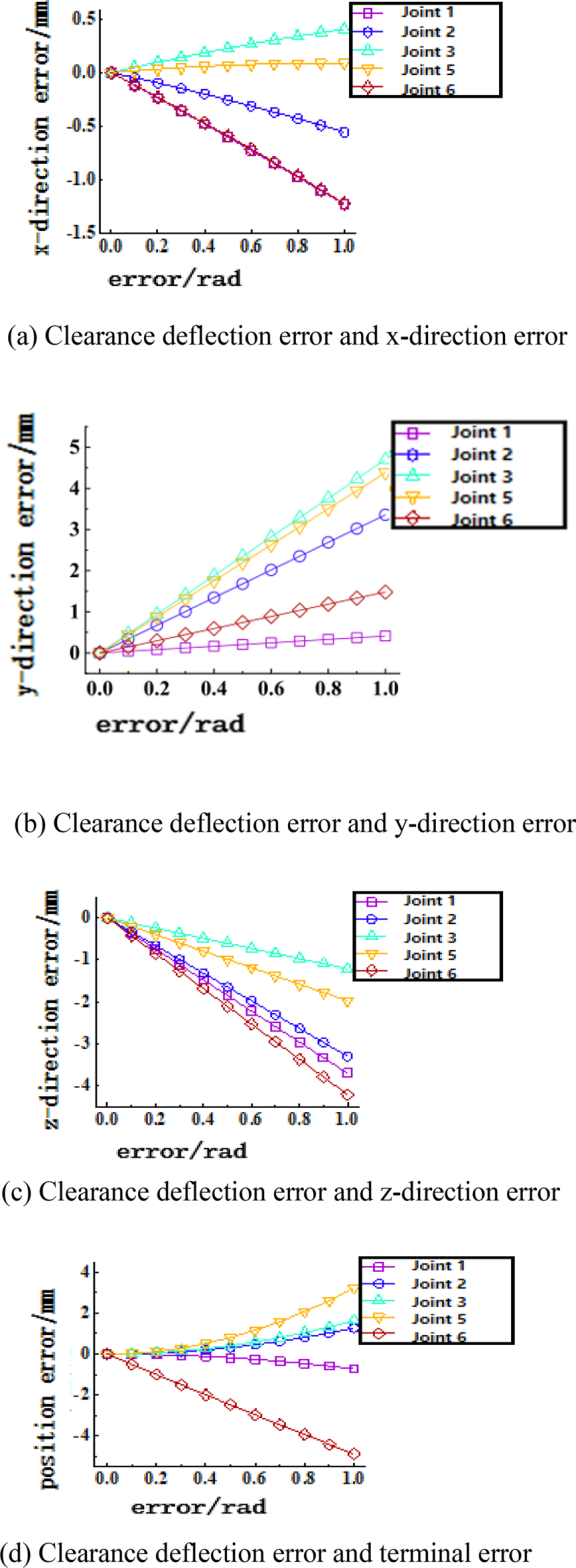
Joint clearance deflection error-terminal position sensitivity curve. (**a**) Clearance deflection error and x-direction error, (**b**) Clearance deflection error and y-direction error, (**c**) Clearance deflection error and z-direction error, (**d**) Clearance deflection error and terminal error.

**Figure 15:**
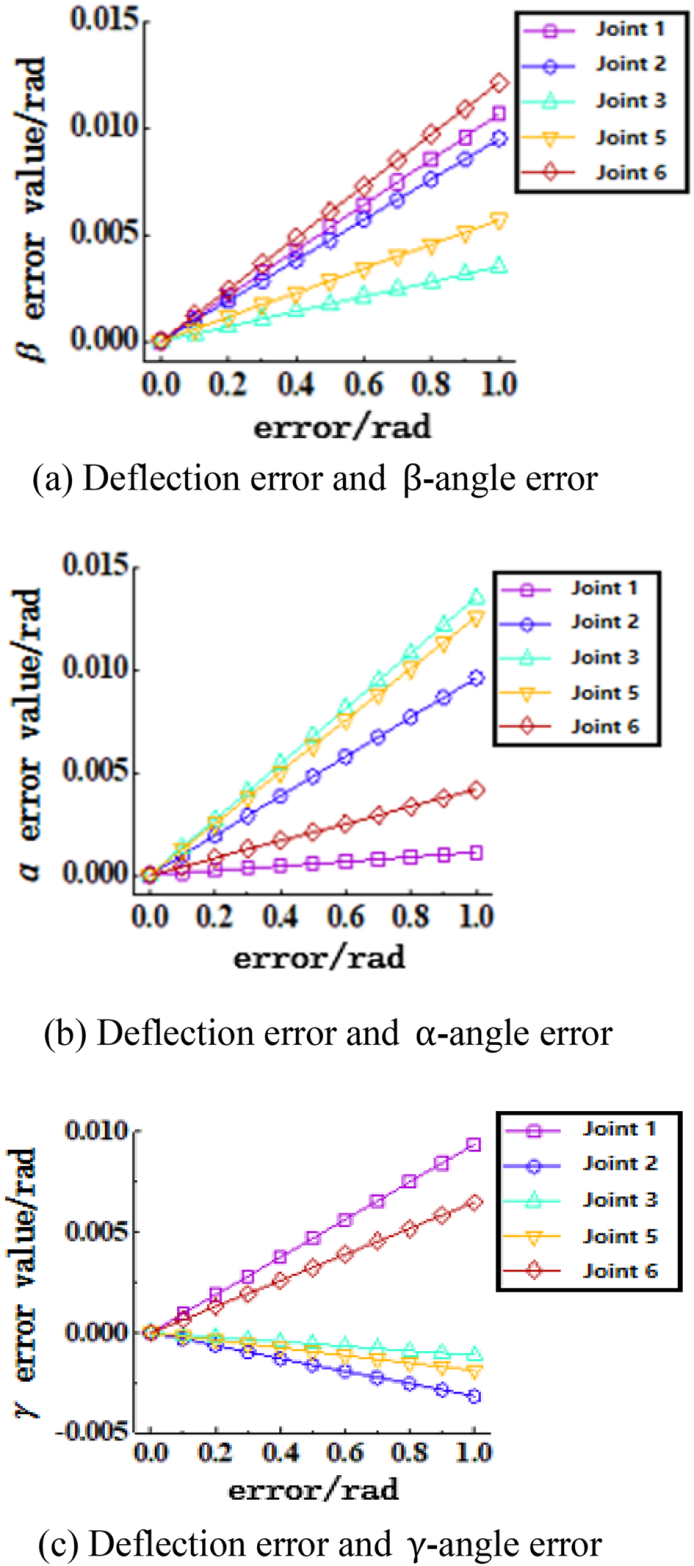
Joint clearance deflection error-terminal attitude angle sensitivity curve, (**a**) Deflection error and $\upbeta$-angle error, (**b**) Deflection error and ${\mathrm{\alpha }}$-angle error, (**c**) Deflection error and $\upgamma$-angle error.

**Figure 16:**
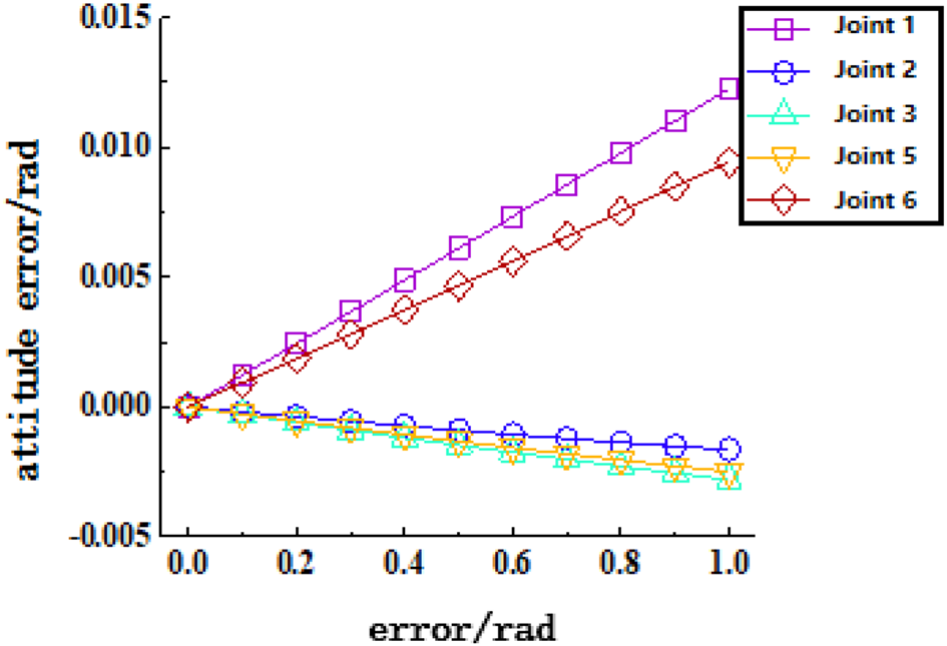
Joint clearance deflection error-terminal attitude sensitivity curve.

Table 5 shows the data characteristics of the terminal pose error under the clearance error. The clearance error has a significant influence on the position error in the x-direction, and the mean value is 1.0033 mm. For the attitude error, the overall effect is not very different. Figures 13, 14, 15 and 16 show the sensitivity analysis of the joint clearance error.

**Table 5:** Distribution characteristics of terminal error.

Dependent variable	Mean value	Standard deviation	Maximum value	Minimum value
$\Delta p/mm$	− 0.2669	0.4281	1.557	− 1.3677
$\Delta x/mm$	1.0033	0.4835	2.9457	− 0.8082
$\Delta y/mm$	5.1599e−04	0.2985	1.0452	− 1.0669
$\Delta z/mm$	− 9.0025e−04	0.3853	1.3884	− 1.3202
$\Delta \varphi/rad$	− 9.9408e−07	2.1801e−04	9.5873e−04	− 9.3875e−04
$\Delta \theta/rad$	6.8450e−07	1.5260e−04	6.5689e−04	− 6.7131e−04
$\Delta \psi/rad$	2.9981e−07	6.9772e−05	3.0010e−04	− 3.0719e−04

Figure 13 shows that the clearance offset error of joints 2, 3, and 5 has the greatest influence on the position error in the x-direction, and the maximum value is 0.394 mm. The clearance offset error of joints 1 and 6 has the greatest influence on the position error in the y-direction, and the maximum value is − 0.263 mm. The clearance offset error of joints 1 and 6 has the greatest influence on the position error in the z-direction, and the maximum value is 0.188 mm. In Fig. 13d, it can be seen that the clearance offset error of joints 2, 3 and 5 have the greatest influence on the terminal position, and the maximum value is 0.395 mm.

Figure 14 shows the sensitivity curve of the joint clearance deflection error to the terminal position. From these four graphs, it can be seen that the clearance error has a nonlinear relationship with the position error. (a) The graph shows that the clearance deflection error of joints 1 and 6 has the greatest influence on the position error in the x-direction, followed by joint 2, and joint 5 has the least influence on the position error in the x-direction. (b) The graph shows that the clearance deflection error of joints 3 and 5 has the greatest influence on the position error in the y-direction, and joint 1 has the least influence on the position error in the y-direction (c) The graph shows that the clearance deflection error of joints 6, 1 and 2 has the greatest influence on the position error in the z-direction, and joint 3 has the least influence on the position error in the z-direction (d) The graph shows that joint 6 has the greatest influence on the terminal position error.

Figure 15 shows the sensitivity curves of the joint clearance deflection error on the attitude angle. (a) The graph shows that joints 6, 1, and 2 have the greatest influence on the error value of attitude angle $\upbeta$, and joint 3 has the least influence. (b) The graph shows that joints 6, 5, and 2 have the greatest influence on the error value of attitude angle $\mathrm{\alpha }$, and joint 1 has the least influence. (c) The graph shows that joints 1 and 6 have the greatest influence on the error value of attitude angle $\upgamma$, and joint 3 has the least influence.

Figure 16 shows the sensitivity curve of the joint clearance deflection error on the terminal posture. In the figure, it can be seen that joints 1 and 6 have the greatest influence on the terminal posture, and the influence of joints 2, 3, and 5 is not very different.

## Verification of numerical simulation

**Figure 17:**
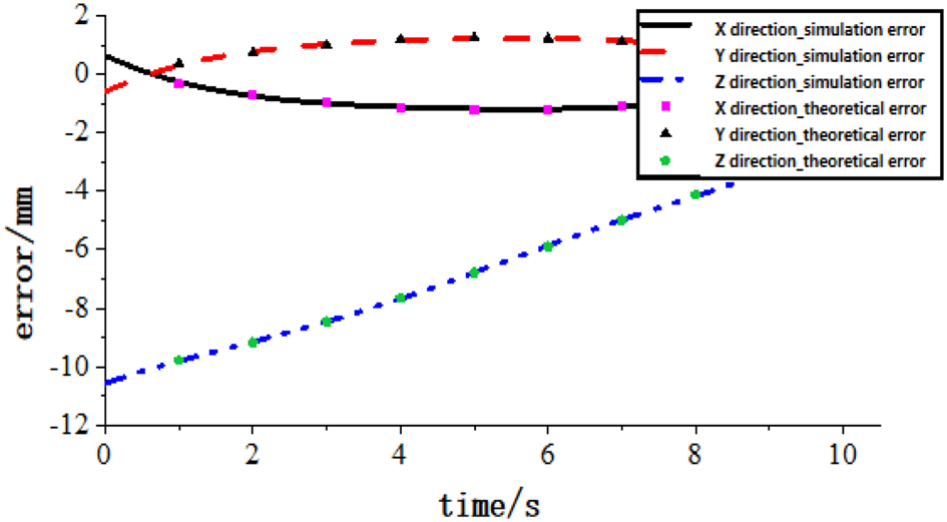
Position error comparison of ADAMS-numerical theory.

To verify the correctness of the rod length-transmission error and clearance error model based on screw theory, the parametric modelling function of ADAMS was used to conduct simulation analysis by building a virtual prototype of the tool-changing robot. MARKER points are established in ADAMS to represent each kind of error. First, the position curves of the terminal in different directions are obtained through the dynamic simulation of the virtual prototype in the ideal state. The terminal position error curves are obtained by the parameterized representation of the error types. The error curves can be obtained by subtracting them. Finally, the error model proposed in this study is used to select multiple groups of specific joint variable values for numerical calculation, and the comparison diagram of position error is obtained, as shown in Fig. 17.

Through comparison, it can be seen that the error model in this study is approximately the same as the results obtained by the ADAMS simulation. Therefore, the model is correct.

**Figure 18:**
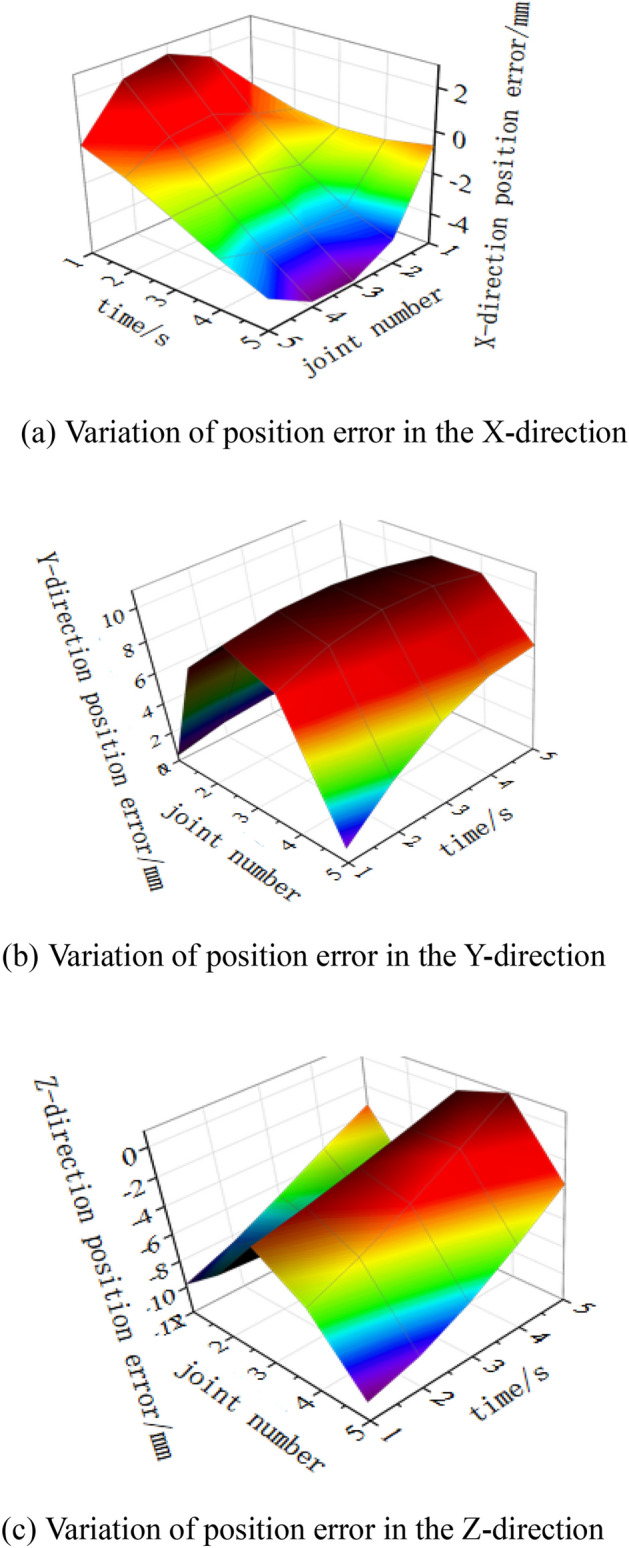
Surface diagram of terminal position error variation. (**a**) Variation of position error in the X-direction, (**b**) Variation of position error in the Y-direction, (**c**) Variation of position error in the Z-direction.

To illustrate the dynamic characteristics of the position errors in different directions under the influence of different operating moments and joint errors, the surface diagram of the terminal position error variation is shown in Fig. 18.

Figure 18 shows that in the operation process, joints 3 and 4 have the greatest influence in the X and Y directions, and the error variable is the largest, especially at the end of the operation. Joints 1 and 5 have the greatest influence in the Z direction, but the influence of joint 2 is greater than that of joint 1 at the later stage of operation.

## Conclusion

In this study, a new modular tool-changing robot is designed and analysed in structural design, working principle, kinematics, and various types of error modelling.

A model of linkage dimensional error and rotation angle error based on screw theory is proposed, and then a numerical analysis is carried out. The linkage parameters significantly influence the pose of the end effector. The results of this paper show that when the allowable error value of the terminal position is 0.25 mm, the motion accuracy is 95.03%.

The error models of axial movement and spatial deflection of joint clearance were established by using screw theory, and the radial movement error in three-dimensional space was analysed by using the double-plane method. For the rotating joints with clearance, the accuracy of each rotating joint clearance and its linkage output position and attitude are analysed and quantified. The results show that the small joint structure parameter error can be mapped to the pose error of the end effector by using screw theory. Therefore, it can improve the accuracy of the error modelling and solve the problem that the D-H method cannot realise the motion along the Y-axis.

## Data Availability

The datasets used and/or analysed during the current study available from the corresponding author on reasonable request.
